# Computational analysis of solar thermal system with Prandtl nanofluid

**DOI:** 10.1038/s41598-022-13845-3

**Published:** 2022-06-21

**Authors:** Muhammad Imran Khan, Muhammad Ijaz Khan, Sami G. Al-Ghamdi

**Affiliations:** 1 Division of Sustainable Development, College of Science & Engineering, Hamad Bin Khalifa University, Qatar Foundation, Doha, Qatar; 2 Nonlinear Analysis and Applied Mathematics (NAAM) Research Group, Department of Mathematics, Faculty of Sciences, King Abdulaziz University, P.O. Box 80203, Jeddah, 21589 Saudi Arabia

**Keywords:** Engineering, Mathematics and computing

## Abstract

The solar thermal system can address a large amount of heating and cooling load required by buildings and industry. To enhance the absorption efficiency in solar thermal systems, nanofluids are considered as promising heat transfer medium. The study presents a numerical study to investigate physical feature of the entropy production in hydro-magnetic reactive unsteady flow of Prandtl nanoliquid over an infinite plate. The heat expression is modeled subject to thermal radiation and magnetic field. Innovative characteristics slip mechanisms i.e., thermophoresis diffusion and Brownian motion are also accounted. Mathematical modeling of entropy production is described by employing thermodynamics law (second law). Furthermore chemical reactions takes place at surface of plate are implemented. Nonlinear system are converted to dimensionless form via suitable transformation. The resultant system is solved by numerical approach (finite difference method). Characteristics of thermal field, entropy rate, fluid flow and concentration are physical discussed through sundry parameters. The outcomes display that the maximum velocity field exists near the center of the surface, whereas the average time flow enhances the velocity distribution. An augmentation in thermal field is distinguished versus magnetic parameter, while reverse behavior holds for fluid flow. An increase in the thermal field with respect to the magnetic variable is noted, while the opposite effect is observed for the fluid flow. A larger approximation of radiation rises entropy rate and thermal field. Increasing the Brownian motion variable increases concentration, while reverse impact is observed for Schmidt number.

## Introduction

Solar thermal system is one of the few scalable technologies capable of delivering dispatch-able renewable power and, as such, many expect it to shoulder a significant share of system balancing in a renewable electricity future power by cheap, intermittent PV and wind power. To efficiently convert the solar radiation into useful heat energy, various technologies are being investigated. Among these, nanofluids are considered as promising heat transfer medium. In recent years, nanofluids have received growing interests by researchers from various fields because of their enhanced thermo-physical properties such as convective heat transfer, viscosity, thermal conductivity and thermal diffusivity. Thanks to these enhanced properties, nanofluids can be used in a wide range of engineering applications, particularly to enhance the thermal performance of solar systems.

This study offers important insights into the 2D unsteady magnetohydrodynamic (MHD) flow with entropy production of nanoliquids subjected to chemical reaction and thermal radiative flux. MHD is the examination of dynamics of magnetic fields of electrically-conducting liquids. The induced current subject to magnetic field in an electrically conducting liquid polarizes the fluid, resulting in a change in the magnetic field. Liquids can be electrically conductive in numerous applications for material processing in both chemical and mechanical engineering and so they react to applied magnetic fields. Such systems can be employed in various industrial applications, for example to monitor the rate of heat transfer levels over a stretch sheet and to get pre-processing materials properties and tuning the thermomechanical processing of materials to industry requirements. This process is very significant subject to Lorentz force, which reduces the liquid flow in a applied field direction.

Investigations of MHD flow of nanofluids with radiative effects are reported by various studies.

Rashad et al.^1^ numerically inspected effects of the sink on MHD flow with entropy production of Cu-water nanoliquid in an inclined permeable enclosure. Their consequences reveals that the heat gradient decreases with swelling the volume fraction of nanoliquid and the intensity of magnetic field. Dharmaiah et al.^2^ deliberated the MHD viscous nanomaterial flow by a stretchable wedge subject to convective conditions, Ohmic heating and radiative heat flux. Izady et al.^3^ investigated CuO/water based nanomaterial flow via permeable expanding surface with MHD and radiative effects. It is revealed that double branch solutions occurs for a certain domain of the surface expanding variable. Dinarvand^4^ scrutinized viscous flow of CuO–Ag/water based nanoliquid over a circular cylinder with sinusoidal radius variation by considering different physical parameters. Jabbaripour et al.^5^ investigated the 3D MHD stagnation-point boundary layer flow of aluminium–copper/water hybrid nanomaterial over a wavy cylinder considering subjected to temperature jump boundary conditions and velocity slip. Mousavi et al.^6^ employs experimental relations to improve model for envisaging the presentation of water-based MHD Casson nanofluid fluid flow over an expanding surface with radiative effect. Some other important recent studies on this topic are listed in Refs.^7–16^.

Entropy production is a innovative prospective in numerous thermodynamic developments and displays dynamic utilizations in polymer processing and thermal optimization. The consequence of entropy production is witnessed in combustion, thermal systems, heat exchangers, turbine systems, nuclear reactions, porous media etc. Thermodynamics second law is employed to discuss the irreversibility analysis. Here a model of entropy generation rate caused by fluid friction, magnetic field effect and solutal transfer rate across a low temperature and concentration difference in the liquid flow is constructed. By minimizing and evaluating the entropy production, the effectiveness of a thermal system can be enhanced, and losses of energy can be minimized. The theoretical analysis of entropy optimization problem in thermal convective flow is investigated by Bejan^17,18^. Kurnia et al.^19^ reported the thermal transport and entropy analyses in viscous flow in helically coiled tubes. Their conclusions showed that the entropy production inside the pipe is higher for thermal transfer than compared to fluid friction. Irreversibility investigation in water-based iron oxide nanofluid with variable magnetic force inside circular tube was interpreted by Gorjaei et al.^20^. Khan et al.^21^ considered melting and irreversibility effects for MHD nanomaterials flow with slip condition. Few advancements regarding entropy problems are mentioned in Refs.^22–30^. Refs.^31–36^ highlights the importance of fluid flow regarding stretchable surfaces.

From above literature review, it shows there are numerous existing studies in the literature pertaining MHD flow of the nanofluids under and boundary conditions and different geometric configurations, however limited studies consider all the effects considered in the present work simultaneously. At the same time, their analysis focuses primarily on local entropy generation. Additionally, no work has been reported so far entropy generation in chemically reactive unsteady flow of Prandtl nanoliquid with Lorentz force over an infinite plate. Therefore in recent communication our prime objective is to analyze the entropy examination in reactive time-dependent flow Prandtl nanomaterials with Lorentz force over an infinite plate. Energy expression is modeled through magnetic force and thermal radiation. Significant behaviors of random and thermophoresis motion are further accounted. Physical features of entropy production are deliberated. Moreover, chemical reaction is also addressed. Nonlinear system are converted to dimensionless system by employing appropriate transformations. The achieved dimensionless problem are tackled through numerical approach (finite difference method). Significant impact of physical variables on fluid flow, entropy generation, thermal field and concentration are discussed via various plots.

## Mathematical description

**Figure 1:**
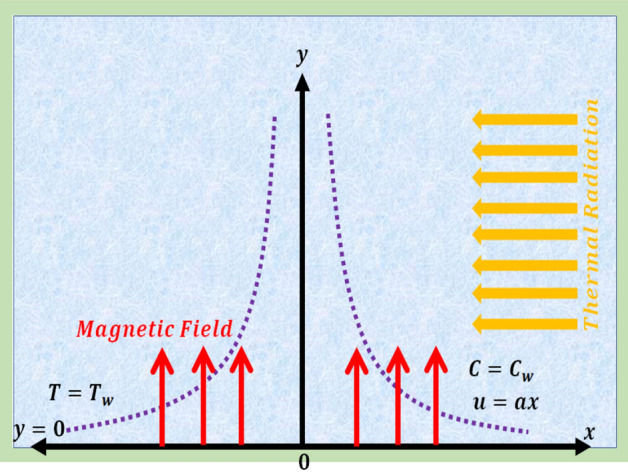
Flow sketch.

Here 2D (two-dimensional) chemically reactive unsteady flow of Prandtl nanofluid with Lorentz force over an infinite plate is discussed. Thermal radiation and magnetic force are considered in energy expression. In addition, innovative characteristics of random and thermophoresis motion are considered. Thermodynamics second law is employed to discuss entropy analysis. Furthermore, chemical reaction at the surface of plate is considered. Constant magnetic force $(B_{0} )$ is applied. Consider $u = u_{w} = ax$ as stretching velocity with ($a > 0$). The schematic flow examination is displayed in Fig. 1.

The extra stress tensor of Prandtl fluid satisfies^37–40^:

1 $$\tau_{ij} = \frac{{A\sinh^{ - 1} \left( {\tfrac{\gamma }{{C_{1} }}} \right)}}{{\left( {\tfrac{\gamma }{{C_{1} }}} \right)}}A_{1}$$

where

2 $$\gamma = \sqrt {\frac{1}{2}tr\left( {A_{1} } \right)^{2} }$$

and

3 $$A_{1} = L + L^{t} ,\,$$

Here $C_{1}$ and $A$ signify the fluid parameters.

The flow equations are

4 $$\frac{\partial u}{{\partial x}} + \frac{\partial v}{{\partial y}} = 0,\,$$

5 $$\frac{\partial u}{{\partial t}} + u\frac{\partial u}{{\partial x}} + v\frac{\partial u}{{\partial y}} = \frac{A}{{\rho_{f} C_{1} }}\frac{{\partial^{2} u}}{{\partial y^{2} }} - \frac{A}{{2\rho_{f} C_{1}^{3} }}\left( {\frac{\partial u}{{\partial y}}} \right)^{2} \frac{{\partial^{2} u}}{{\partial y^{2} }} - \frac{{\sigma_{f} B_{0}^{2} }}{{\rho_{f} }}u,\,$$

6 $$\left. {\begin{array}{*{20}c} {\tfrac{\partial T}{{\partial t}} + u\tfrac{\partial T}{{\partial x}} + v\tfrac{\partial T}{{\partial y}} = \tfrac{{k_{f} }}{{\left( {\rho c_{p} } \right)_{f} }}\left( {1 + \tfrac{{16\sigma^{ * } T_{\infty }^{3} }}{{3kk^{ * } }}} \right)\,\tfrac{{\partial^{2} T}}{{\partial y^{2} }} + \tau D_{B} \tfrac{\partial T}{{\partial y}}\tfrac{\partial C}{{\partial y}}} \\ { + \tau \tfrac{{D_{T} }}{{T_{\infty } }}\left( {\tfrac{\partial T}{{\partial y}}} \right)^{2} + \tfrac{{\sigma B_{0}^{2} }}{{\left( {\rho c_{p} } \right)_{f} }}u^{2} } \\ \end{array} } \right\}\,$$

7 $$\frac{\partial C}{{\partial t}} + u\frac{\partial C}{{\partial x}} + v\frac{\partial C}{{\partial y}} = D_{B} \frac{{\partial^{2} C}}{{\partial y^{2} }} + \frac{{D_{T} }}{{T_{\infty } }}\frac{{\partial^{2} T}}{{\partial y^{2} }} - k_{r} \left( {C - C_{\infty } } \right)\,$$

with,

8 $$\left. {\begin{array}{*{20}c} {u = 0,\, \, v = 0,\,\;T = T_{\infty } ,\,\;C = C_{\infty } \;\;{\text{at }}t = 0} \\ {u = ax{, }v = 0,\,\;T = T_{w} ,\,\;C = C_{w} \;\;{\text{at }}y = 0} \\ {u = 0,\, \, v = 0,\, \, T = T_{\infty } {, }C = C_{\infty } {\text{ as }}y \to \infty } \\ \end{array} } \right\}\,$$

Let us consider

9 $$\left. {\begin{array}{*{20}c} {\tau = \tfrac{\nu }{{L_{1}^{2} }}t{, }\xi = \tfrac{x}{{L_{1} }}{, }\eta = \tfrac{y}{{L_{1} }}{, }U\left( {\tau ,\,\xi ,\,\eta } \right) = \tfrac{{L_{1} }}{\nu }u{, }V\left( {\tau ,\,\xi ,\,\eta } \right) = \tfrac{{L_{1} }}{\nu }v} \\ {\theta \left( {\tau ,\,\xi ,\,\eta } \right) = \tfrac{{T - T_{\infty } }}{{T_{w} - T_{\infty } }}{, }\phi \left( {\tau ,\,\xi ,\,\eta } \right) = \tfrac{{C - C_{\infty } }}{{C_{w} - C_{\infty } }}} \\ \end{array} } \right\},\,$$

We get

10 $$\frac{\partial U}{{\partial \xi }} + \frac{\partial V}{{\partial \eta }} = 0,\,$$

11 $$\frac{\partial U}{{\partial \tau }} + U\frac{\partial U}{{\partial \xi }} + V\frac{\partial U}{{\partial \eta }} = \alpha \frac{{\partial^{2} U}}{{\partial \eta^{2} }} - \beta \left( {\frac{\partial U}{{\partial \eta }}} \right)^{2} \frac{{\partial^{2} U}}{{\partial \eta^{2} }} - {\text{Re}} MU,\,$$

12 $$\frac{\partial \theta }{{\partial \tau }} + U\frac{\partial \theta }{{\partial \xi }} + V\frac{\partial \theta }{{\partial \eta }} = \frac{1}{\Pr }\left( {1 + Rd} \right)\,\frac{{\partial^{2} \theta }}{{\partial \eta^{2} }} + Nb\frac{\partial \theta }{{\partial \eta }}\frac{\partial \phi }{{\partial \eta }} + Nt\left( {\frac{\partial \theta }{{\partial \eta }}} \right)^{2} + MEcU^{2} ,\,$$

13 $$\frac{\partial \phi }{{\partial \tau }} + U\frac{\partial \phi }{{\partial \xi }} + V\frac{\partial \phi }{{\partial \eta }} = \frac{{\partial^{2} \phi }}{{\partial \eta^{2} }} + \frac{Nt}{{Nb}}\frac{{\partial^{2} \theta }}{{\partial \eta^{2} }} - Sc\lambda \phi ,\,$$

subject to

14 $$\left. {\begin{array}{*{20}c} {U = 0{, }V = 0{, }\theta = 0{, }\phi = 0{\text{ at }}\tau = 0} \\ {U = Re\xi {, }V = 0{, }\theta = 1{, }\phi = 1{\text{ at }}\eta = 0} \\ {U = 0{, }V = 0{, }\theta = 0{, }\phi = 0{\text{ as }}\eta \to \infty } \\ \end{array} } \right\}.$$

In above expression the dimensionless variables are $M\left( { = \tfrac{{\sigma_{f} B_{0}^{2} }}{{a\rho_{f} }}} \right),$ $\alpha \left( { = \tfrac{A}{{\mu_{f} C_{1} }}} \right),$ $Re\left( { = \tfrac{{aL_{1}^{2} }}{{\nu_{f} }}} \right),$ $\beta \left( { = \tfrac{{A\nu_{f} }}{{2\rho_{f} C_{1}^{3} L_{1}^{4} }}} \right),$ $\Pr \left( { = \tfrac{{\mu_{f} c_{p} }}{{k_{f} }}} \right),$ $Nt\left( { = \tfrac{{\tau D_{T} \left( {T_{w} - T_{\infty } } \right)}}{{\nu_{f} T_{\infty } }}} \right)$, $Rd\left( { = \tfrac{{16\sigma^{ * } T_{\infty }^{3} }}{{3k^{ * } k_{f} }}} \right),$ $Nb\left( { = \tfrac{{\tau D_{B} \left( {C_{w} - C_{\infty } } \right)}}{{\nu_{f} }}} \right)$, $Br\left( { = \Pr Ec} \right),$ $Sc\left( { = \tfrac{{\nu_{f} }}{{D_{B} }}} \right)$ and $\delta \left( { = \tfrac{{k_{r} L_{1}^{2} }}{{\nu_{f} }}} \right)$.

## Entropy generation

It is defined as

15 $$\left. {\begin{array}{*{20}c} {E_{G} = \tfrac{k}{{T_{\infty }^{2} }}\left( {1 + \tfrac{{16\sigma^{ * } T_{\infty }^{3} }}{{3k^{ * } k_{f} }}} \right)\,\left( {\tfrac{\partial T}{{\partial y}}} \right)^{2} + \tfrac{{\sigma B_{0}^{2} }}{{T_{\infty } }}u^{2} + \tfrac{{RD_{B} }}{{T_{\infty } }}\tfrac{\partial T}{{\partial y}}\tfrac{\partial C}{{\partial y}}} \\ { + \tfrac{{RD_{B} }}{{C_{\infty } }}\left( {\tfrac{\partial C}{{\partial y}}} \right)^{2} } \\ \end{array} } \right\},\,$$

Finally we can found

16 $$S_{G} (\tau ,\,\zeta ,\,\eta ) = \alpha_{1} \left( {1 + Rd} \right)\,\left( {\frac{\partial \theta }{{\partial \eta }}} \right)^{2} + MBrU^{2} + L\frac{\partial \theta }{{\partial \eta }}\frac{\partial \phi }{{\partial \eta }} + L\frac{{\alpha_{2} }}{{\alpha_{1} }}\left( {\frac{\partial \phi }{{\partial \eta }}} \right)^{2} .$$

Here dimensionless parameters are $\alpha_{1} \left( { = \tfrac{{\left( {T_{w} - T_{\infty } } \right)}}{{T_{\infty } }}} \right),$ $S_{G} \left( { = \tfrac{{E_{G} T_{\infty } L_{1}^{2} }}{{k_{f} \left( {T_{w} - T_{\infty } } \right)}}} \right),$ $\alpha_{2} \left( { = \tfrac{{\left( {C_{w} - C_{\infty } } \right)}}{{C_{\infty } }}} \right)$ and $L\left( { = \tfrac{{RD_{B} \left( {C_{w} - C_{\infty } } \right)}}{{k_{f} }}} \right)$.

## Solution methodology

The dimensionless partial systems are solved by numerical approach (Finite difference method). Finite difference methods for dimensionless partial systems are expressed as^41–43^:

17 $$\left. {\begin{array}{*{20}c} {\tfrac{\partial U}{{\partial \tau }} = \tfrac{{U_{n,\,m}^{p + 1} - U_{n,\,m}^{p} }}{\Delta \;\tau },\, \, \tfrac{\partial U}{{\partial \xi }} = \tfrac{{U_{n + 1,\,m}^{p} - U_{n,\,m}^{p} }}{\Delta \;\xi },\, \, \tfrac{\partial U}{{\partial \eta }} = \tfrac{{U_{n,\,m + 1}^{p} - U_{n,\,m}^{p} }}{\Delta \;\eta }} \\ {\tfrac{\partial V}{{\partial \tau }} = \tfrac{{V_{n,\,m}^{p + 1} - V_{n,\,m}^{p} }}{\Delta \;\tau },\, \, \tfrac{\partial V}{{\partial \xi }} = \tfrac{{V_{n + 1,\,m}^{p} - V_{n,\,m}^{p} }}{\Delta \;\xi },\, \, \tfrac{\partial V}{{\partial \eta }} = \tfrac{{V_{n,\,m + 1}^{p} - V_{n,\,m}^{p} }}{\Delta \;\eta }} \\ {\tfrac{\partial \theta }{{\partial \tau }} = \tfrac{{\theta_{n,\,m}^{p + 1} - \theta_{n,\,m}^{p} }}{\Delta \;\tau },\, \, \tfrac{\partial \theta }{{\partial \xi }} = \tfrac{{\theta_{n + 1,\,m}^{p} - \theta_{n,\,m}^{p} }}{\Delta \;\xi },\, \, \tfrac{\partial \theta }{{\partial \eta }} = \tfrac{{\theta_{n,\,m + 1}^{p} - \theta_{n,\,m}^{p} }}{\Delta \;\eta }} \\ {\tfrac{\partial \phi }{{\partial \tau }} = \tfrac{{\phi_{n,\,m}^{p + 1} - \phi_{n,\,m}^{p} }}{\Delta \;\tau },\, \, \tfrac{\partial \phi }{{\partial \xi }} = \tfrac{{\phi_{n + 1,\,m}^{p} - \phi_{n,\,m}^{p} }}{\Delta \;\xi },\, \, \tfrac{\partial \phi }{{\partial \eta }} = \tfrac{{\phi_{n,\,m + 1}^{p} - \phi_{n,\,m}^{p} }}{\Delta \;\eta }} \\ {\tfrac{{\partial^{2} U}}{{\partial \xi^{2} }} = \tfrac{{U_{n + 2,\,m}^{p} - 2U_{n + 1,\,m}^{p} + U_{n,\,m}^{p} }}{{\left( {\Delta \;\xi } \right)^{2} }},\, \, \tfrac{{\partial^{2} U}}{{\partial \eta^{2} }} = \tfrac{{U_{n,\,m + 2}^{p} - 2U_{n,\,m + 1}^{p} + U_{n,\,m}^{p} }}{{\left( {\Delta \;\eta } \right)^{2} }}} \\ {\tfrac{{\partial^{2} V}}{{\partial \xi^{2} }} = \tfrac{{V_{n + 2,\,m}^{p} - 2V_{n + 1,\,m}^{p} + V_{n,\,m}^{p} }}{{\left( {\Delta \;\xi } \right)^{2} }},\, \, \tfrac{{\partial^{2} V}}{{\partial \eta^{2} }} = \tfrac{{V_{n,\,m + 2}^{p} - 2V_{n,\,m + 1}^{p} + V_{n,\,m}^{p} }}{{\left( {\Delta \;\eta } \right)^{2} }}} \\ {\tfrac{{\partial^{2} \theta }}{{\partial \xi^{2} }} = \tfrac{{\theta_{n + 2,\,m}^{p} - 2\theta_{n + 1,\,m}^{p} + \theta_{n,\,m}^{p} }}{{\left( {\Delta \;\xi } \right)^{2} }},\, \, \tfrac{{\partial^{2} \theta }}{{\partial \eta^{2} }} = \tfrac{{\theta_{n,\,m + 2}^{p} - 2\theta_{n,\,m + 1}^{p} + \theta_{n,\,m}^{p} }}{{\left( {\Delta \;\eta } \right)^{2} }}} \\ {\tfrac{{\partial^{2} \phi }}{{\partial \xi^{2} }} = \tfrac{{\phi_{n + 2,\,m}^{p} - 2\phi_{n + 1,\,m}^{p} + \phi_{n,\,m}^{p} }}{{\left( {\Delta \;\xi } \right)^{2} }},\, \, \tfrac{{\partial^{2} \phi }}{{\partial \eta^{2} }} = \tfrac{{\phi_{n,\,m + 2}^{p} - 2\phi_{n,\,m + 1}^{p} + \phi_{n,\,m}^{p} }}{{\left( {\Delta \;\eta } \right)^{2} }}} \\ \end{array} } \right\}\,$$

Using Eq. (17) in Eqs. (10–14) we get

18 $$\frac{{U_{n + 1,\,m}^{p} - U_{n,\,m}^{p} }}{\Delta \;\xi } + \frac{{V_{n,\,m + 1}^{p} - V_{n,\,m}^{p} }}{\Delta \;\eta } = 0,\,$$

19 $$\left. {\begin{array}{*{20}c} {\tfrac{{U_{n,\,m}^{p + 1} - U_{n,\,m}^{p} }}{\Delta \;\tau } + U_{n,\,m}^{p} \tfrac{{U_{n + 1,\,m}^{p} - U_{n,\,m}^{p} }}{\Delta \;\xi } + V_{n,\,m}^{p} \tfrac{{U_{n,\,m + 1}^{p} - U_{n,\,m}^{p} }}{\Delta \;\eta } = \alpha \tfrac{{U_{n,\,m + 2}^{p} - 2U_{n,\,m + 1}^{p} + U_{n,\,m}^{p} }}{{\left( {\Delta \;\eta } \right)^{2} }}} \\ { - \beta \left( {\tfrac{{U_{n,\,m + 1}^{p} - U_{n,\,m}^{p} }}{\Delta \;\eta }} \right)^{2} \tfrac{{U_{n,\,m + 2}^{p} - 2U_{n,\,m + 1}^{p} + U_{n,\,m}^{p} }}{{\left( {\Delta \;\eta } \right)^{2} }} - {\text{Re}} MU_{n,\,m}^{p} } \\ \end{array} } \right\},\,$$

20 $$\left. {\begin{array}{*{20}c} {\tfrac{{\theta_{n,\,m}^{p + 1} - \theta_{n,\,m}^{p} }}{\Delta \;\tau } + U_{n,\,m}^{p} \tfrac{{\theta_{n + 1,\,m}^{p} - \theta_{n,\,m}^{p} }}{\Delta \;\xi } + V_{n,\,m}^{p} \tfrac{{\theta_{n,\,m + 1}^{p} - \theta_{n,\,m}^{p} }}{\Delta \;\eta } = \tfrac{1}{\Pr }\left( {1 + Rd} \right)\,\tfrac{{\theta_{n,\,m + 2}^{p} - 2\theta_{n,\,m + 1}^{p} + \theta_{n,\,m}^{p} }}{{\left( {\Delta \;\eta } \right)^{2} }}} \\ {Nb\left( {\tfrac{{\theta_{n,\,m + 1}^{p} - \theta_{n,\,m}^{p} }}{\Delta \;\eta }} \right)\,\left( {\tfrac{{\phi_{n,\,m + 1}^{p} - \phi_{n,\,m}^{p} }}{\Delta \;\eta }} \right) + Nt\left( {\tfrac{{\theta_{n,\,m + 1}^{p} - \theta_{n,\,m}^{p} }}{\Delta \;\eta }} \right)^{2} + MEc\left( {U_{n,\,m}^{p} } \right)^{2} } \\ \end{array} } \right\},\,$$

21 $$\left. {\begin{array}{*{20}c} {\tfrac{{\phi_{n,\,m}^{p + 1} - \phi_{n,\,m}^{p} }}{\Delta \;\tau } + U_{n,\,m}^{p} \tfrac{{\phi_{n + 1,\,m}^{p} - \phi_{n,\,m}^{p} }}{\Delta \;\xi } + V_{n,\,m}^{p} \tfrac{{\phi_{n,\,m + 1}^{p} - \phi_{n,\,m}^{p} }}{\Delta \;\eta } = \tfrac{{\phi_{n,\,m + 2}^{p} - 2\phi_{n,\,m + 1}^{p} + \phi_{n,\,m}^{p} }}{{\left( {\Delta \;\eta } \right)^{2} }}} \\ {\tfrac{Nt}{{Nb}}\left( {\tfrac{{\theta_{n,\,m + 2}^{p} - 2\theta_{n,\,m + 1}^{p} + \theta_{n,\,m}^{p} }}{{\left( {\Delta \;\eta } \right)^{2} }}} \right) - Sc\lambda \phi_{n,\,m}^{p} } \\ \end{array} } \right\}\,$$

with

22 $$\left. {\begin{array}{*{20}c} {U_{n,\,m}^{0} = 0,\, \, V_{n,\,m}^{0} = 0,\, \, \theta_{n,\,m}^{0} = 0,\, \, \phi_{n,\,m}^{0} = 0} \\ {U_{n,\,0}^{p} = Re\left( {\xi_{n + 1} - \xi_{n} } \right),\, \, V_{n,\,0}^{p} = 0,\, \, \theta_{n,\,0}^{p} = 1,\, \, \phi_{n,\,0}^{p} = 1} \\ {U_{n,\infty }^{p} = 0,\, \, V_{n,\infty }^{p} = 0,\, \, \theta_{n,\infty }^{p} = 0,\, \, \phi_{n,\infty }^{p} = 0} \\ \end{array} } \right\}.$$

Entropy generation satisfy

23 $$\left. {\begin{array}{*{20}c} {S_{G} = \alpha_{1} \left( {1 + Rd} \right)\,\left( {\tfrac{{\theta_{n,\,m + 1}^{p} - \theta_{n,\,m}^{p} }}{\Delta \;\eta }} \right)^{2} + MBr\left( {U_{n,\,m}^{p} } \right)^{2} } \\ { + L\tfrac{{\theta_{n,\,m + 1}^{p} - \theta_{n,\,m}^{p} }}{\Delta \;\eta }\tfrac{{\phi_{n,\,m + 1}^{p} - \phi_{n,\,m}^{p} }}{\Delta \;\eta } + L\tfrac{{\alpha_{2} }}{{\alpha_{1} }}\left( {\tfrac{{\phi_{n,\,m + 1}^{p} - \phi_{n,\,m}^{p} }}{\Delta \;\eta }} \right)^{2} } \\ \end{array} } \right\}.$$

## Discussion

Noteworthy presentation of fluid flow, entropy generation, concentration and thermal field against physical variables are graphically scrutinized.

### Velocity

**Figure 2:**
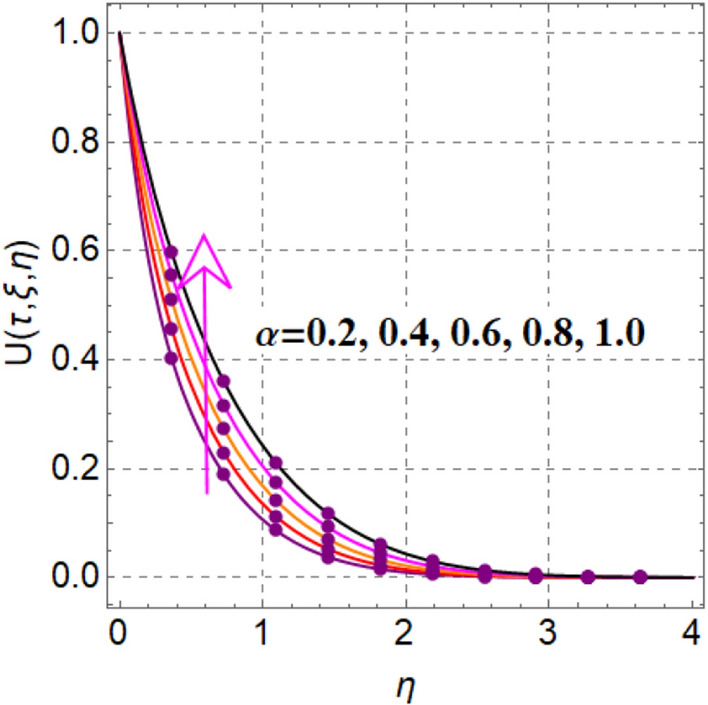
$$U\left( {\tau ,\,\xi ,\,\eta } \right)$$ via $$\alpha$$.

**Figure 3:**
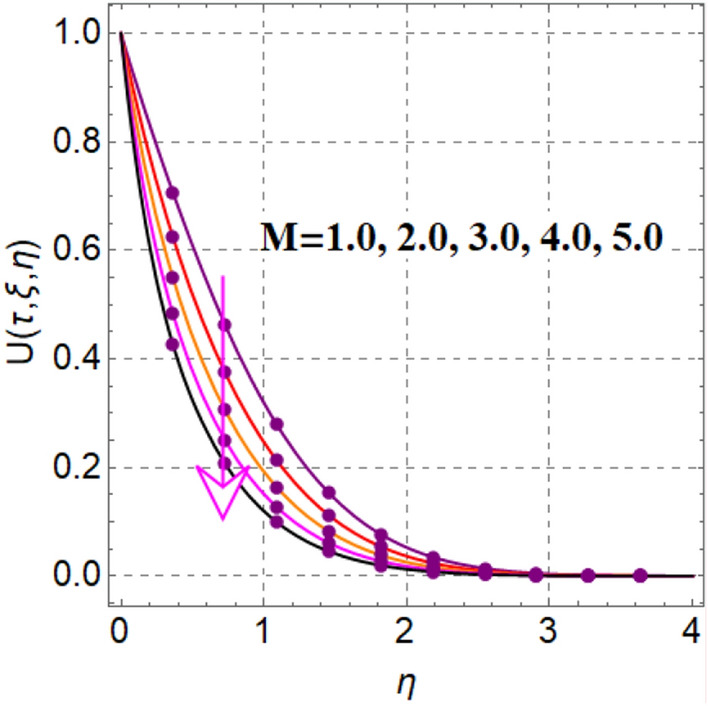
$$U\left( {\tau ,\,\xi ,\,\eta } \right)$$ via $$M$$.

Significant effect of fluid parameter on velocity is illustrated in Fig. 2. Clearly velocity is augmented for fluid variable. Physically higher approximation of fluid parameter decreases viscosity, which augments fluid flow. Figure 3 outcomes impact of magnetic variable on velocity. Here velocity is decreased for magnetic variable. This decreasing behavior is because of Lorentz force.

### Temperature

**Figure 4:**
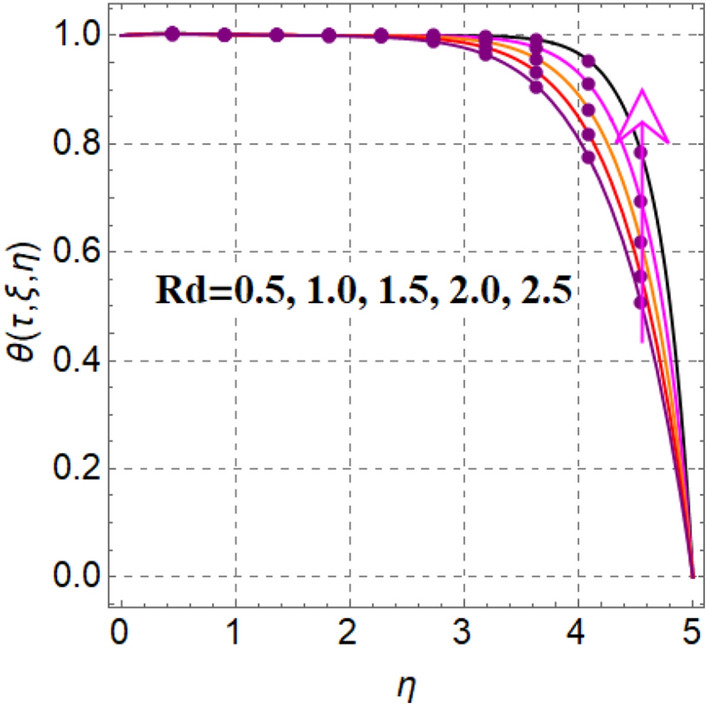
$$\theta \left( {\tau ,\,\xi ,\,\eta } \right)$$ via $$Rd$$.

**Figure 5:**
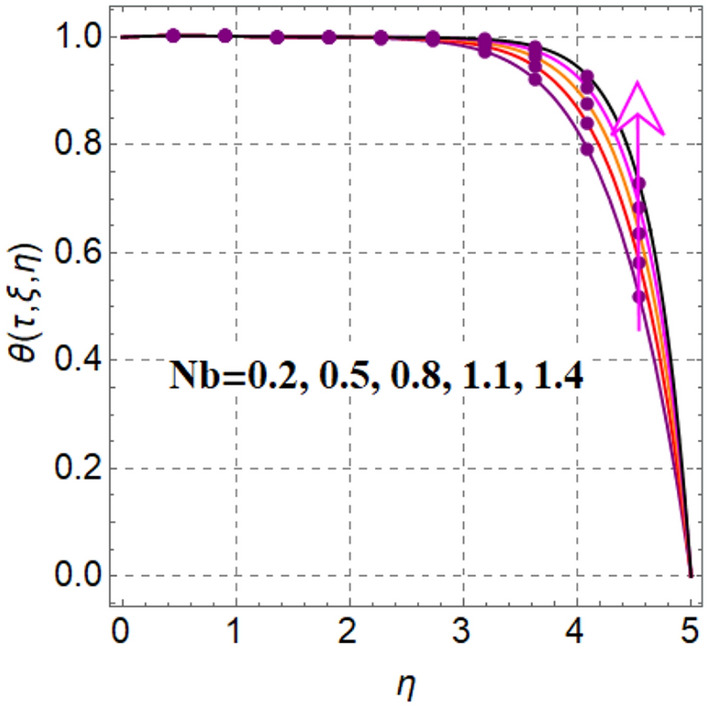
$$\theta \left( {\tau ,\,\xi ,\,\eta } \right)$$ via $$Nb$$.

**Figure 6:**
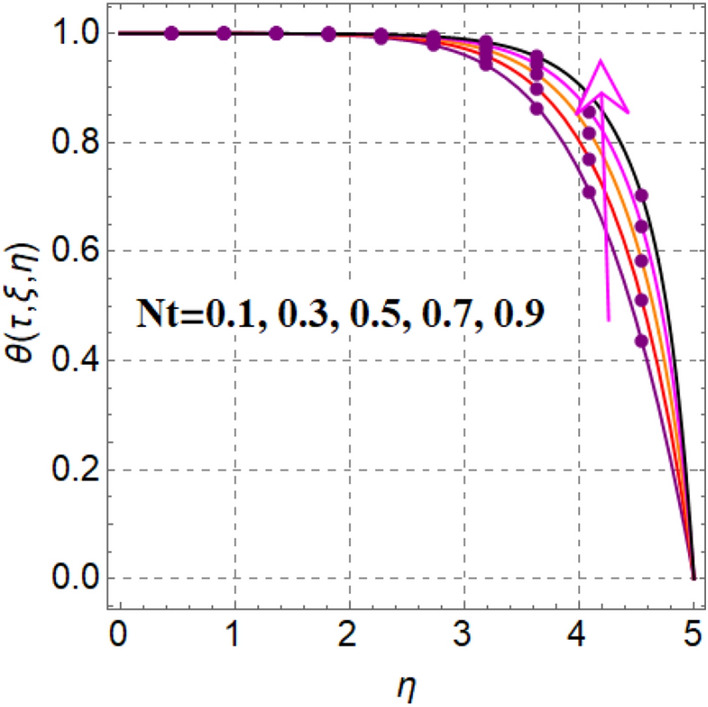
$$\theta \left( {\tau ,\,\xi ,\,\eta } \right)$$ via $$Nt$$.

**Figure 7:**
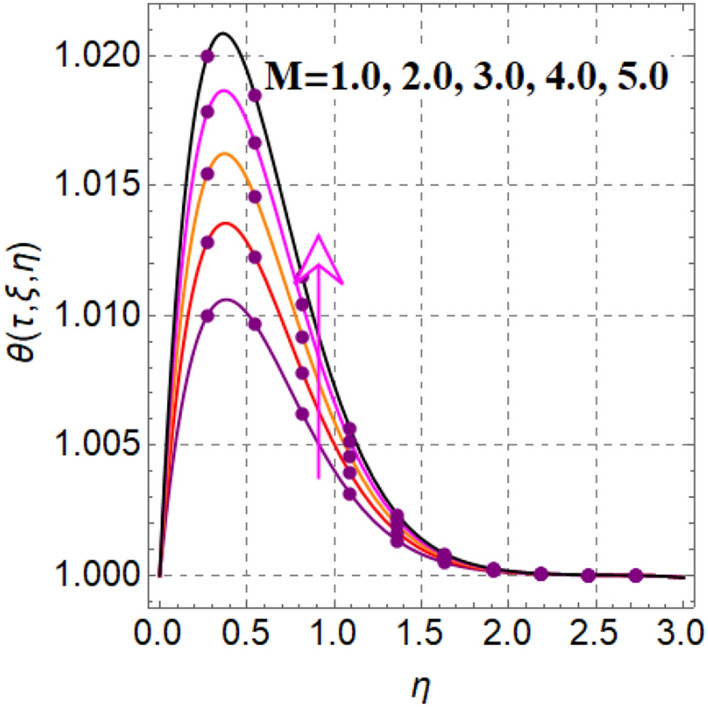
$$\theta \left( {\tau ,\,\xi ,\,\eta } \right)$$ via $$M$$.

Salient feature of radiation on thermal field is portrayed in Fig. 4. It is renowned that temperature augments via radiation. Figure 5 captured inspiration of random motion parameter on thermal field. Here thermal field increased through random motion variable. Figure 6 is sketched to see thermal field performance versus thermophoresis variable. One can find that temperature boosted with variation in thermophoresis effect. In fact increasing values of thermophoresis variable generates a force corresponds to nanoparticles from warm region to cold region. As a result thermal field boosted. Figure 7 is intrigued to see influence of magnetic variable on thermal field. An intensification in resistive force with variation in magnetic variable, which enhances collision between liquid particles. Therefore thermal field is augmented.

### Concentration

**Figure 8:**
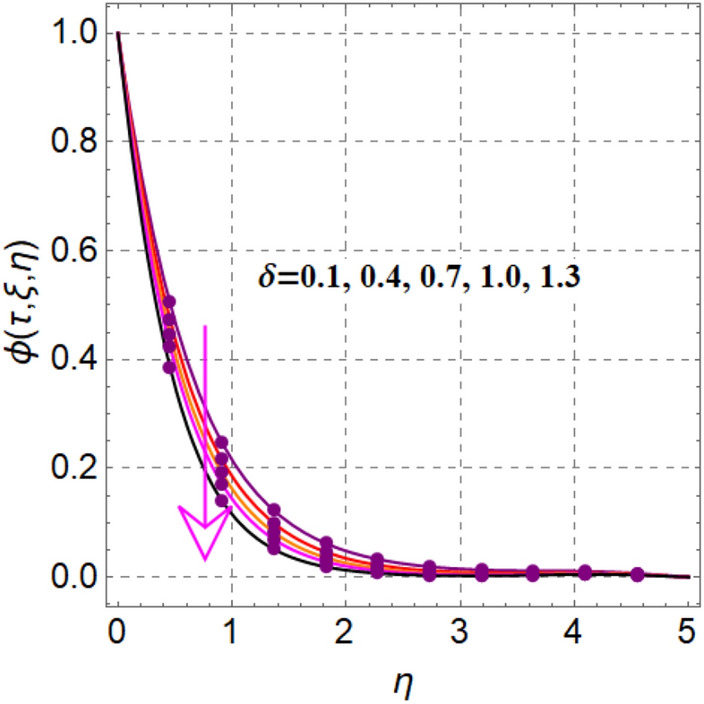
$$\phi \left( {\tau ,\,\xi ,\,\eta } \right)$$ via $$\delta$$.

**Figure 9:**
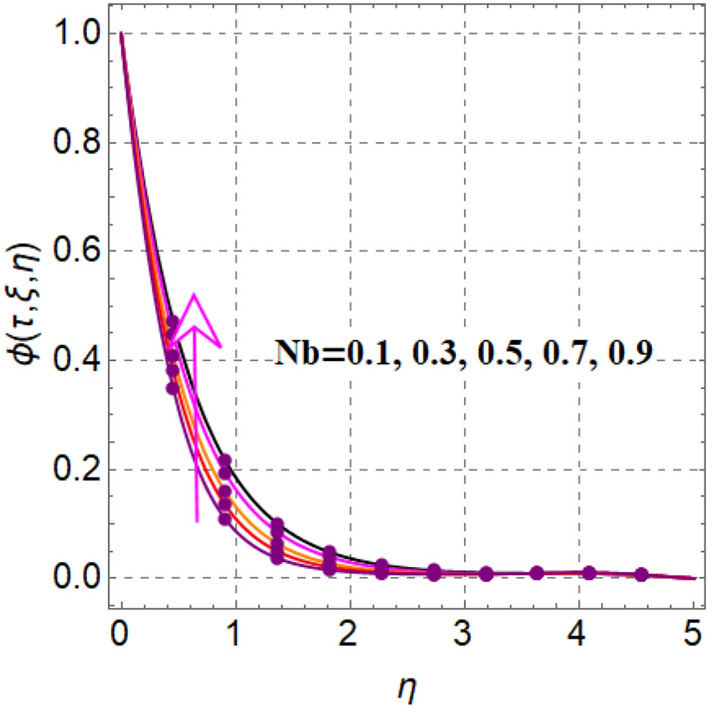
$$\phi \left( {\tau ,\,\xi ,\,\eta } \right)$$ via $$Nb$$.

**Figure 10:**
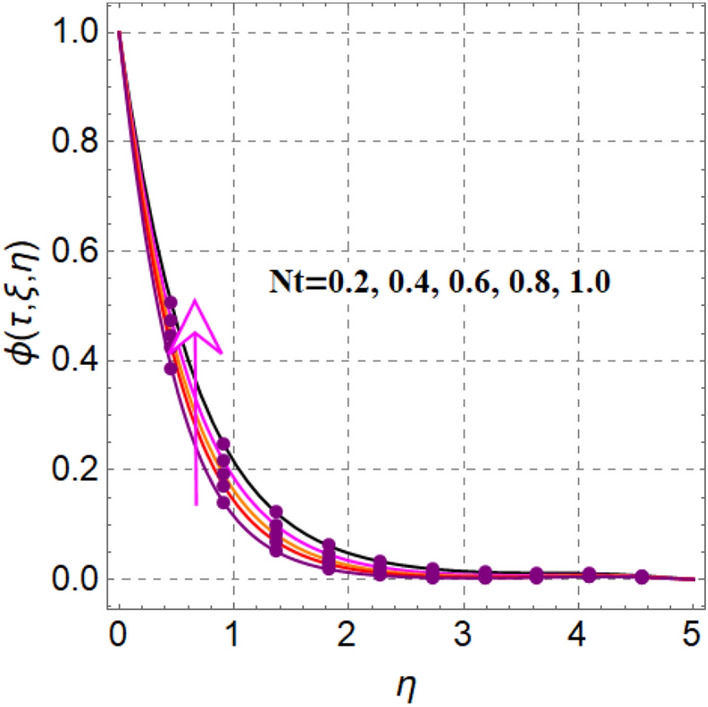
$$\phi \left( {\tau ,\,\xi ,\,\eta } \right)$$ via $$Nt$$.

**Figure 11:**
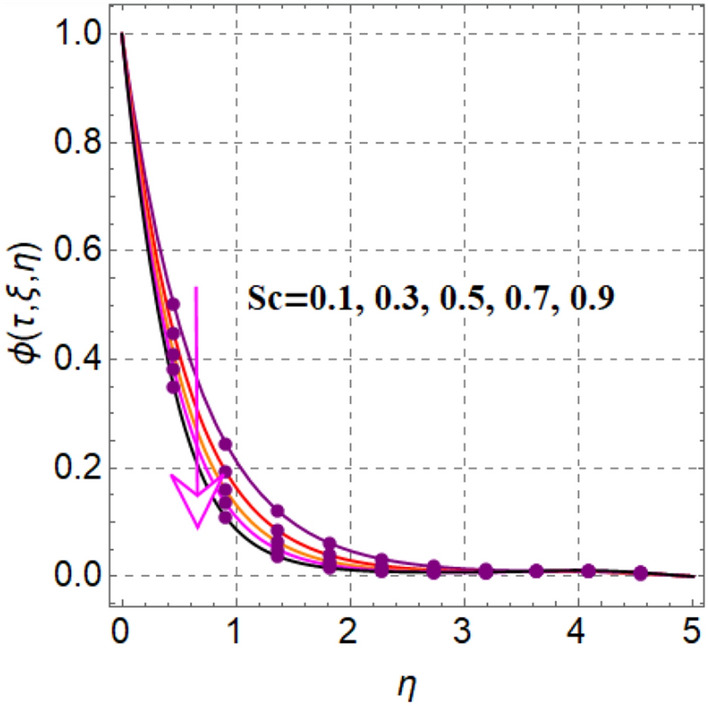
$$\phi \left( {\tau ,\,\xi ,\,\eta } \right)$$ via $$Sc$$.

Figure 8 elaborates the performance of concentration versus reaction variable ($\delta$). Here concentration decays with rising values of reaction variable ($\delta$). Significant features of concentration against random and thermophoresis variables ($Nb$ and $Nt$) are drafted in Figs. 9 and 10. Clearly an intensification in concentration is noted through random and thermophoretic variables ($Nb$ and $Nt$). Figure 11 reflects outcomes of concentration via Schmidt number. Larger estimation of Schmidt number decays diffusion behaviors and as a result concentration decreased.

### Entropy generation

**Figure 12:**
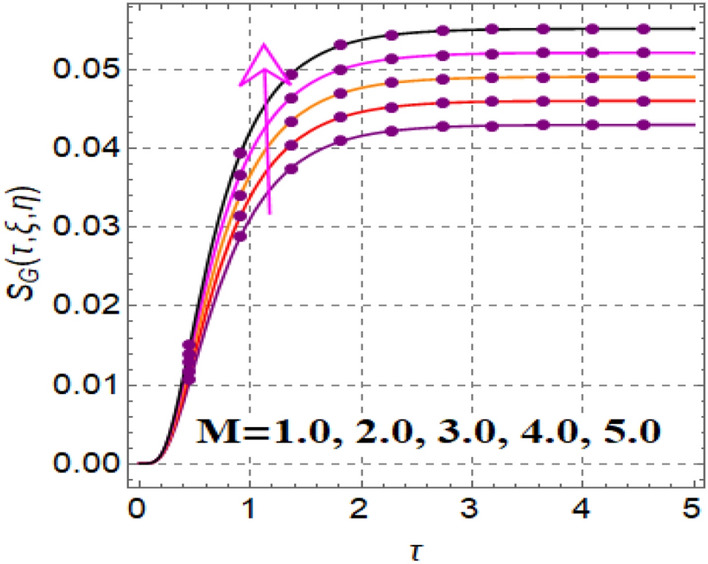
$$S_{G} \left( {\tau ,\,\xi ,\,\eta } \right)$$ via $$M$$.

**Figure 13:**
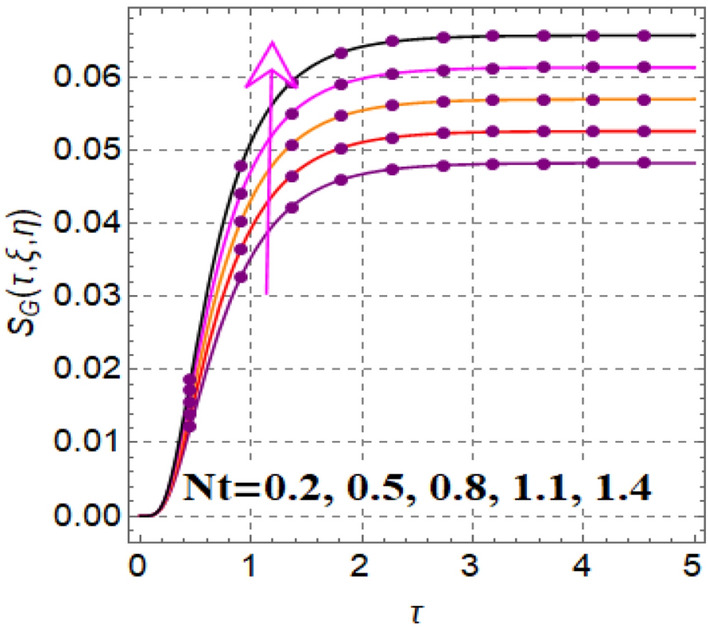
$$S_{G} \left( {\tau ,\,\xi ,\,\eta } \right)$$ via $$Nt$$.

**Figure 14:**
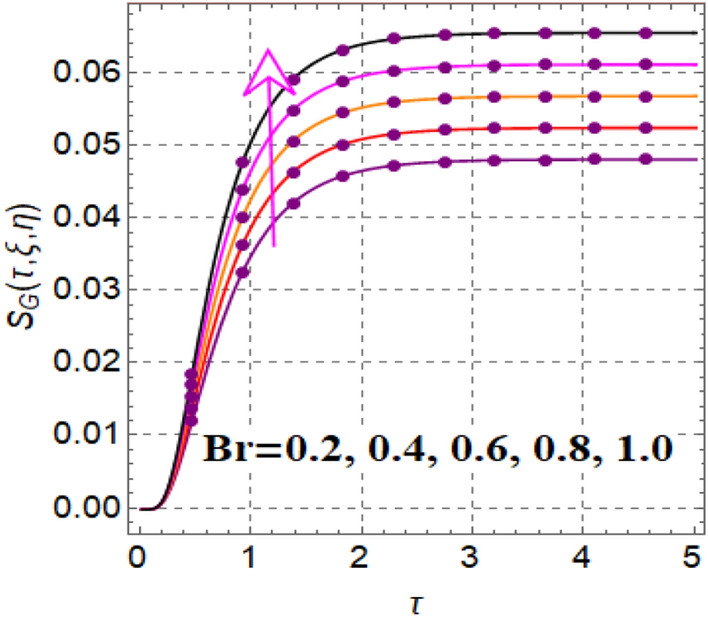
$$S_{G} \left( {\tau ,\,\xi ,\,\eta } \right)$$ via $$Br$$.

**Figure 15:**
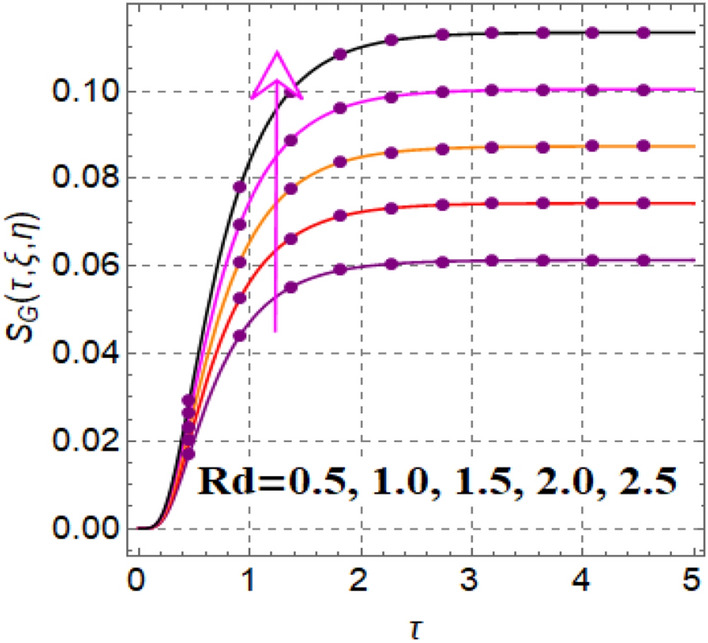
$$S_{G} \left( {\tau ,\,\xi ,\,\eta } \right)$$ via $$Rd$$.

Figure 12 shows significance of magnetic variable on entropy production. A development in Lorentz force is noticed subject to magnetic variable, which augments disorderness in thermal system. As a outcome entropy generation is increased. Influence of thermophoretic variable on entropy rate is illuminated in Fig. 13. Clearly entropy optimization boosts up versus thermophoretic variable. Influence of entropy generation via Brinkman number is elucidated in Fig. 14. Larger ($Br$) improves the entropy rate. The consequences of radiation factor on entropy profile is displayed in Fig. 15. An intensification occurs in entropy rate with variation in radiation effect.

## Final remarks

Key main conclusions of present flow problem are displayed as:

- Velocity field is declined against magnetic field, while opposite effect holds for thermal profile.
- Larger fluid variable improves fluid flow.
- An intensification in radiation boosts up entropy rate and thermal field.
- An amplification in temperature is seen subject to slip mechanisms i.e., Brownian motion and thermophoretic diffusion.
- An opposite impact holds for concentration through reaction and random motion variable.
- A decrement in concentration is noticed for Schmidt number.
- A similar effect holds for entropy production and concentration through thermophoresis variable.
- An amplification in entropy production is seen subject to magnetic variable and Brinkman number.

## Data Availability

All data generated during this study are included in this published article.

## List of symbols

$$u,\,v$$: Velocity components
$$x,\,y$$: Cartesian coordinates
$$t$$: Time
$a$: Stretching rate constant
$B_{0}$: Magnetic field strength
$\tau_{ij}$: Extra stress tensor
$$A,\,C_{1}$$: Fluid variables
$$\rho_{f}$$: Density
$$\mu_{f}$$: Dynamic viscosity
$$\nu_{f}$$: Kinematic viscosity
$$\sigma_{f}$$: Electrical conductivity
$$T$$: Temperature
$$T_{w}$$: Wall temperature
$$T_{\infty }$$: Ambient temperature
$$k_{f}$$: Thermal conductivity
$$c_{p}$$: Specific heat
$\sigma^{ * }$: Stefan Boltzmann constant
$k^{ * }$: Mean absorption coefficient
$\tau$: Ratio of heat capacities
$$D_{T}$$: Thermophoresis coefficient
$$C$$: Concentration
$$C_{w}$$: Wall concentration
$$C_{\infty }$$: Ambient concentration
$$k_{r}$$: Reaction rate
$R$: Molar gas constant
$$M$$: Magnetic parameter
$\alpha ,\,\beta$: Prandtl fluid variables
$$Re$$: Reynold number
$$\Pr$$: Prandtl number
$$Nt$$: Thermophoresis variable
$$Rd$$: Radiation variable
$Nb$: Brownian motion variable
$$\delta$$: Reaction parameter
$$Sc$$: Schmidt number
$$Br$$: Brinkman number
$$S_{G}$$: Entropy rate
$\alpha_{1}$: Temperature ratio variable
$$L$$: Diffusion variable
$\alpha_{2}$: Concentration ratio variable
